# Elevated homocysteine level in first-episode schizophrenia patients—the relevance of family history of schizophrenia and lifetime diagnosis of cannabis abuse

**DOI:** 10.1007/s11011-014-9534-3

**Published:** 2014-03-30

**Authors:** Blazej Misiak, Dorota Frydecka, Ryszard Slezak, Patryk Piotrowski, Andrzej Kiejna

**Affiliations:** 1 Department of Psychiatry, Wroclaw Medical University, 10 Pasteur Street, 50-367 Wroclaw, Poland; 2 Department of Genetics, Wroclaw Medical University, 1 Marcinkowski Street, 50-368 Wroclaw, Poland

**Keywords:** One-carbon metabolism, Homocysteine, Vitamin B12, Folate, Cannabis, First-episode schizophrenia

## Abstract

Accumulating evidence indicates that elevated homocysteine (Hcy) level occurs in first-episode schizophrenia (FES) patients. We included 56 FES patients and 53 healthy controls (HC). Plasma level of Hcy was significantly higher in FES patients than HC (*p* = 0.044). In addition, plasma levels of high-density lipoproteins (HDL) and folate were significantly lower in FES than in HC (*p* < 0.001). Positive family history of schizophrenia was associated with lower plasma HDL (*p* = 0.041) and vitamin B12 (*p* = 0.017), as well as higher level of Hcy (*p* = 0.017). Patients with FES, who abused cannabis, had higher levels of Hcy (*p* = 0.017), as well as lower levels of vitamin B12 (*p* = 0.017) and HDL (*p* = 0.041). Plasma Hcy negatively correlated with duration of untreated psychosis (*r* = −0.272, *p* = 0.042). There was a positive correlation between Hcy level and the severity of negative symptoms (*r* = 0.363, *p* = 0.006) and general psychopathology (*r* = 0.349, *p* = 0.008) assessed using Positive and Negative Syndrome Scale (PANSS). Vitamin B12 level was negatively associated with the severity of negative symptoms (*r* = −0.406, *p* = 0.002), while folate level negatively correlated with general psychopathology score (*r* = −0.365, *p* = 0.006) in PANSS. These results indicate that the severity of one-carbon metabolism alterations and HDL deficiency might be associated with family history of schizophrenia and cannabis abuse. Lower vitamin B12 and folate along with elevated Hcy may influence the severity of FES psychopathology.

## Introduction

Re-methylation of homocysteine (Hcy) to methionine constitutes one of the most important reactions catalysed in one-carbon metabolism. There are numerous factors that influence Hcy level including age, gender, cigarette smoking, alcohol consumption, various medications and polymorphisms in genes encoding enzymes acting in one-carbon metabolism (Nygard et al. 1995; de Bree et al. 2001; Dierkes et al. 2007). Two single nucleotide polymorphisms (C677T and A1298C) in the methylenetetrahydrofolate (*MTHFR*) gene are also important genetic predictors of Hcy level (Ulrich et al. 2002).

Hyperhomocysteinemia is widely recognized as the risk factor for cardiovascular complications. Accumulating evidence indicates that Hcy level is also increased in schizophrenia patients (Misiak et al. 2013). Inconsistent results indicate that elevated Hcy level occurs in first-episode schizophrenia (FES) patients. It has also been found that a 5-μmol/l increase of plasma Hcy may increase the risk of schizophrenia by 70 % (Muntjewerff et al. 2006). Additionally, Hcy level may positively correlate with duration of untreated psychosis (DUP) (Ayesa-Arriola et al. 2012), the severity of negative symptoms (Goff et al. 2004; Petronijevic et al. 2008; Bouaziz et al. 2010) and cognitive deficits (Levine et al. 2006). Some authors suggest that mainly young schizophrenia males demonstrate elevated Hcy levels (Levine et al. 2002). In the recent study by Geller et al. (2013), it was found that siblings of schizophrenia patients are also characterized by elevated Hcy level. These findings are in line with previous studies showing the association between polymorphisms in the *MTHFR* gene and schizophrenia (Muntjewerff et al. 2006). Polymorphisms in the *MTHFR* gene might also predict the development of metabolic side effects associated with second-generation antipsychotics (Misiak et al. 2013). In addition, extensive evidence indicates that the prevalence of lipid and glucose metabolism disturbances is significantly higher in first-episode and drug-naïve schizophrenia patients in comparison with healthy controls (Chen et al. 2013; Wu et al. 2013; Cai et al. 2012; Spelman et al. 2007; Ryan et al. 2003; McEvoy et al. 2013). These findings point to the hypothesis that schizophrenia is in itself associated with metabolic deregulation.

A number of environmental factors including alcohol consumption, cigarette smoking and various medications may influence one-carbon metabolism (Nygard et al. 1995; de Bree et al. 2001; Dierkes et al. 2007). There are also studies showing low folate level due to cannabis abuse in pregnant women (Knight et al. 1994) and undergraduate students (Davis et al. 1978). However, the influence of cannabis on plasma levels of Hcy, vitamin B12 and folate has not been investigated so far. Cannabis abuse is increasingly recognized as a risk factor for the development of psychotic disorders including those from schizophrenia spectrum [for review see (Casadio et al. 2011)]. In the register-based study of 18,478 Finnish inpatients with first hospitalization due to cannabis-induced psychosis, the 8-year cumulative risk of schizophrenia spectrum diagnosis was estimated at 46 % (Niemi-Pynttari et al. 2013). Notably, cannabis may exert long-term deleterious effects. For instance, the longitudinal study of 45,570 Swedish conscripts revealed that 31 % of cannabis users, who later developed schizophrenia, stopped using cannabis before the day of conscription (Zammit et al. 2002).

Several mechanisms may underlie the relationship between Hcy and schizophrenia. It has been shown that Hcy administered in high concentration is an agonist at the glutamate binding site and a partial antagonist at the glycine co-agonist site in NMDA receptors (Lipton et al. 1997). These findings may be in line with studies showing that hypofunction of glutamatergic transmission acts in the pathophysiology of schizophrenia (Coyle et al. 2012). Furthermore, Hcy may initiate neuronal apoptosis (Wang et al. 2012; Fujiki et al. 2012), trigger mitochondrial dysfunction (Kumar et al. 2011) and promote oxidative stress (Dietrich-Muszalska et al. 2012).

Although there is considerable interest in one-carbon metabolism alterations in schizophrenia, some questions still remain. There is a scarcity of studies on first-episode and/or drug-naïve schizophrenia patients (Garcia-Bueno et al. 2013; Ayesa-Arriola et al. 2012; Kale et al. 2010; Bicikova et al. 2011). These studies have also provided contradictory results. Therefore, it is still unresolved as to whether increased Hcy level is associated with schizophrenia or occurs due to antipsychotic treatment. Furthermore, given the limited efficacy of folate supplementation in the treatment of negative symptoms (Hill et al. 2011; Roffman et al. 2013b), it seems that searching for clinical variables associated with Hcy level alterations, which may predict the outcome of supplementation strategies, is warranted.

The aim of this study was to bridge the gaps in studies on one-carbon metabolism in schizophrenia and explore whether family history of schizophrenia and cannabis abuse influence plasma levels of Hcy, vitamin B12 and folate in FES patients.

## Material and methods

### Subjects

We recruited 56 FES inpatients and 53 healthy controls (HC) with negative family history of schizophrenia or other psychotic disorders. Both groups were matched for age, gender, body mass index (BMI) and ethnicity (all participants were Caucasians). All subjects provided written informed consent for participation in the study. Exclusion criteria were: mental retardation and/or general brain disorder, supplementation of folic acid or vitamins B, positive urine screening for illicit drugs (cannabis, amphetamine, opiates and ecstasy), drug and/or alcohol abuse/dependence during 1 year prior to the onset of psychotic symptoms, severe somatic comorbidities, the use of statins, fibrates, anti-hypertensive drugs or anti-diabetic medications and inability to give informed consent. A diagnosis of schizophrenia was based on DSM-IV and ICD-10 criteria and confirmed using the Operational Criteria for Psychotic Illness (OPCRIT) checklist. OPCRIT constitutes a modern diagnostic tool creating a multidimensional insight into the course and psychopathology of psychotic disorders (McGuffin et al. 1991). Furthermore, OPCRIT has high reliability determined using a kappa statistic in several classification systems e.g. kappa for ICD-10 and DSM-III-R has been estimated at 0.70 and 0.73 respectively (Williams et al. 1996). We included the patients, who met DSM-IV and ICD-10 criteria for lifetime diagnosis of cannabis abuse, but who reported cannabis cessation at least 1 year before the onset of schizophrenia. In addition, psychopathology was examined by using the Positive and Negative Syndrome Scale (PANSS) (Kay et al. 1987). DUP was defined as the time from appearance of the first prodromal symptoms to initiation of antipsychotic treatment. Assessment of psychopathology and diagnosing processes were based on structured interviews with the patients and on medical records. Clinical assessment with OPCRIT checklist and PANSS was performed by a trained and experienced clinician (B.M.). Cigarette smoking was evaluated using pack-year index and the Fagerström test (Pomerleau et al. 1989). Notably, OPCRIT was completed for the lifetime course of the disorder and evaluation of psychopathology with PANSS was performed on the day of recruitment. All patients were examined up to 16 days since the admission day. Patients had a minimal dose of second-generation antipsychotics (olanzapine in 21 patients, risperidone in 21 patients and amisulpride in 2 patients) on the day of assessment and there were 12 drug-naïve patients. Agitation and hostility were managed with haloperidol and benzodiazepines. No other psychotropic medications were used. Average treatment duration was 5.10 ± 4.45 days, while the average chlorpromazine equivalent was 150 ± 137.8 mg per day on the day of recruitment.

### Metabolic parameters

Blood samples were obtained between 7.30 and 8.30 a.m. after at least 10-h overnight fasting from the antecubital vein. Plasma glucose, lipoproteins, vitamin B12, folate and total cholesterol (TC) were determined using a Cobas 6000 analyzer (Roche, Switzerland). Enzymatic assay of hexokinase was applied to measure plasma glucose. In addition, enzymatic assays of esterase and cholesterol oxidase were used to measure TC level. High-density lipoprotein (HDL) level was measured using polyethylene glycol-modified enzymes. Similarly, enzymatic methods with phosphoglycerol oxidase and peroxidase were used to measure plasma triglycerides. Plasma low-density lipoproteins (LDL) were calculated using the Friedewald equation (Friedewald et al. 1972): LDL (mg/dl) = TC – (HDL + TG/5). Plasma Hcy level was measured using a chemiluminescence method in an Immulite 2000 analyzer (Siemens, Germany). In addition, elektrochemiluminescence method was used to measure plasma folate and vitamin B12.

Weight and height were determined using a balance beam scale with stadiometer, which was located on a firm and horizontal surface. All subjects wore light clothing and stood straight without shoes. Body mass index (BMI) was calculated by dividing body mass in kilograms by the square of the height in meters (kg/m^2^).

### Statistics

Demographic and clinical data between FES patients and HC, between patients with positive and negative history of schizophrenia, as well as between patients with and without lifetime diagnosis of cannabis abuse were compared using the Mann–Whitney *U*-test (age, DUP, BMI, biochemical parameters, pack-year index, Fagerström test score, treatment duration, chlopromazine equivalent, PANSS subscales) and *χ* ^2^ test (gender, the number of cigarette smokers). Differences were considered as statistically significant if the *p* value was <0.05. Correlations between biochemical parameters and illness duration, age of onset of cannabis use, pack-year index and Fagerström test score were assessed using the Spearman’s rank correlation coefficient. Partial Bonferroni correction (Gong et al. 2000), which takes into account correlations between studied variables, was applied to the level of significance due to multiple comparisons of several metabolic parameters that are not independent. Multivariable linear regression model was performed to determine the independent predictors of Hcy level in FES patients. The model included: age, gender, BMI, pack-year index, CPZ equivalent, treatment duration, as well as plasma levels of folate, vitamin B12, LDL, HDL, TG and TC. All analyses were performed using the Statistical Package for Social Sciences (SPSS) version 20.

## Results

The comparison of FES patients and healthy controls (HC) is presented in Table 1. Mean Hcy level was significantly higher (*p* = 0.044) in FES patients (12.38 ± 5.76 μmol/l) in comparison with HC (11.42 ± 6.97 μmol/l). In addition, mean folate and HDL levels were significantly lower (*p* < 0.001) in FES patients (6.32 ± 2.85 ng/ml and 51.62 ± 16.19 mg/dl, respectively) in comparison with HC (11.10 ± 15.20 ng/ml and 66.07 ± 17.46 mg/dl, respectively). These differences cannot be attributed to differences in age, gender and BMI. Notably, mean pack-year index and the Fagerström test score were significantly higher (*p* = 0.038 and *p* = 0.022, respectively) in FES patients (2.09 ± 3.66 and 1.91 ± 2.79, respectively) in comparison with HC (1.01 ± 2.40 and 0.83 ± 1.79, respectively). However, there was no correlation between biochemical parameters and the indices of cigarette smoking (Table 2). Similarly, treatment duration and chlorpromazine equivalent did not correlate with biochemical parameters (Table 2). There were no significant gender differences in Hcy, folate and vitamin B12 levels neither in FES patients nor in HC (data not shown). The linear regression model revealed that after controlling for confounding variables, Hcy levels in FES patients were associated only with folate levels (*B* = −1.10, 95CI% −1.65 to −0.56, *p* < 0.0001).

**Table 1:** The comparison of first-episode schizophrenia patients and healthy controls

	FES patients (*N* = 56)	HC (*N* = 53)	*p*-value^*^
Age (years)	27.19 ± 7.30	25.68 ± 2.89	0.869
Gender (males/females)	30/26	26/27	0.703
BMI (kg/m^2^)	23.62 ± 3.92	22.40 ± 2.95	0.178
Glucose (mg/dl)	85.72 ± 9.43	86.74 ± 10.83	0.121
LDL (mg/dl)	93.16 ± 31.81	85.68 ± 24.65	0.317
HDL (mg/dl)	51.62 ± 16.19	66.07 ± 17.46	**<0.001** ^†^
TC (mg/dl)	168.21 ± 37.63	170.81 ± 30.89	0.727
TG (mg/dl)	112.67 ± 61.25	95.11 ± 45.42	0.123
Hcy (μmol/l)	12.38 ± 5.76	11.42 ± 6.97	**0.044**
Folate (ng/ml)	6.32 ± 2.85	11.10 ± 15.20	**<0.001** ^†^
Vitamin B12 (pg/ml)	399.21 ± 202.47	388.72 ± 146.78	0.671
The number of cigarette smokers	21	10	**0.036**
Pack-year index	2.09 ± 3.66	1.01 ± 2.40	**0.038**
Fagerström test	1.91 ± 2.79	0.83 ± 1.79	**0.022**

*Abbreviations*: *BMI* body mass index, *FES* first-episode schizophrenia, *HC* healthy controls, *Hcy* homocysteine, *HDL* high density lipoproteins, *LDL* low density lipoproteins, *TC* total cholesterol, *TG* triglycerides

^*^ *p*-value refers to the comparison of mean-ranks using the Mann–Whitney *U*-test. Significant differences (*p* < 0.05) were marked in bold

^†^Significant differences after application of partial Bonferroni correction (*p* < 0.0058)

**Table 2:** Correlation coefficients for confounders associated with antipsychotic treatment and cigarette smoking (*p* > 0.05)

	FES patients			HC		
	Pack-year index	Fagerström score	Chlorpromazine equivalent	Treatment duration	Pack-year index	Fagerström score
Glucose	*r* = −0.070	*r* = −0.078	*r* = −0.058	*r* = −0.048	*r* = 0.025	*r* = 0.023
LDL	*r* = 0.195	*r* = 0.145	*r* = 0.122	*r* = −0.034	*r* = 0.243	*r* = 0.237
HDL	*r* = −0.090	*r* = −0.101	*r* = −0.143	*r* = −0.100	*r* = −0.007	*r* = −0.010
TC	*r* = 0.180	*r* = 0.125	*r* = 0.023	*r* = −0.094	*r* = 0.184	*r* = 0.186
TG	*r* = 0.004	*r* = −0.016	*r* = 0.045	*r* = −0.164	*r* = 0.094	*r* = 0.105
Hcy	*r* = 0.058	*r* = 0.062	*r* = −0.049	*r* = −0.136	*r* = 0.037	*r* = 0.040
Folate	*r* = −0.134	*r* = −0.168	*r* = −0.045	*r* = 0.099	*r* = 0.065	*r* = 0.073
Vitamin B12	*r* = 0.006	*r* = 0.028	*r* = 0.064	*r* = 0.189	*r* = −0.083	*r* = −0.062

*Abbreviations*: *BMI* body mass index, *FES* first-episode schizophrenia, *HC* healthy controls, *Hcy* homocysteine, *HDL* high density lipoproteins, *HyperHcy* hyperhomocysteinemia, *LDL* low density lipoproteins, *TC* total cholesterol, *TG* triglycerides

Table 3 presents a comparison of FES patients with and without positive family history of schizophrenia. Patients with a positive family history of schizophrenia in first and/or second degree relatives had significantly higher mean levels of Hcy in comparison with those with a negative family history of schizophrenia (13.40 ± 6.35 μmol/l vs. 9.86 ± 2.74 μmol/l, *p* = 0.017). In addition, mean plasma levels of HDL and vitamin B12 were significantly lower in FES subjects (*p* = 0.041 and *p* = 0.017, respectively), who had first and/or second degree relatives with schizophrenia (49.04 ± 15.60 mg/dl and 350.24 ± 142.98 pg/ml, respectively) in comparison with those, who did not have (58.10 ± 16.28 mg/dl and 521.64 ± 273.45 pg/ml, respectively). There were no between group differences with respect to family history of schizophrenia in age, gender, BMI, psychopathological manifestation assessed by PANSS, the Fagerström test score and pack-year index.

**Table 3:** The comparison of first-episode schizophrenia patients with respect to family history of schizophrenia

	Patients with positive family history of schizophrenia (*N* = 16)	Patients with negative family history of schizophrenia (*N* = 40)	*p*-value^*^
Age (years)	26.07 ± 6.68	30.00 ± 8.23	0.102
Gender (males/females)	7/9	23/17	0.388
BMI (kg/m^2^)	24.16 ± 3.95	22.29 ± 3.66	0.091
Glucose (mg/dl)	86.10 ± 9.39	84.76 ± 9.77	0.599
LDL (mg/dl)	94.85 ± 32.43	88.93 ± 30.76	0.479
HDL (mg/dl)	49.04 ± 15.60	58.10 ± 16.28	**0.041**
TC (mg/dl)	168.43 ± 39.21	167.69 ± 34.56	0.913
TG (mg/dl)	116.23 ± 62.25	103.75 ± 59.69	0.272
Hcy (μmol/l)	13.40 ± 6.35	9.86 ± 2.74	**0.017**
Folate (ng/ml)	6.17 ± 2.89	6.68 ± 2.81	0.425
Vitamin B12 (pg/ml)	350.24 ± 142.98	521.64 ± 273.45	**0.017**
The number of cigarette smokers	5	16	0.761
Pack-year index	1.93 ± 3.11	2.46 ± 4.87	0.746
Fagerström test	2.00 ± 2.76	1.68 ± 2.93	0.678
PANSS—positive symptoms score	23.50 ± 3.29	23.47 ± 5.97	0.793
PANSS—negative symptoms score	18.00 ± 7.73	17.67 ± 7.38	0.806
PANSS—general psychopathology score	41.00 ± 6.78	43.22 ± 8.05	0.342

*Abbreviations*: *BMI* body mass index, *Hcy* homocysteine, *HDL* high density lipoproteins, *LDL* low density lipoproteins, *PANSS* the Positive and Negative Syndrome Scale, *TC* total cholesterol, *TG* triglycerides

^*^ *p*-value refers to the comparison of mean-ranks using the Mann–Whitney *U*-test. Significant differences (*p* < 0.05) were marked in bold. There was no significant difference after application of partial Bonferroni correction (*p* > 0.0055)

We divided FES patients into two subgroups—those with a lifetime diagnosis of cannabis abuse and those, who had never abused cannabis. Patients with a lifetime diagnosis of cannabis abuse had significantly lower mean levels of plasma HDL (45.83 ± 18.56 mg/dl vs. 55.10 ± 18.56, *p* = 0.041) and vitamin B12 (382.48 ± 134.25 pg/ml vs. 409.25 ± 235.44, *p* = 0.017) in comparison with those, who had not abused cannabis (Table 4). On the other hand, lifetime diagnosis of cannabis abuse was associated with significantly higher mean level of plasma Hcy (14.85 ± 7.35 μmol/l vs. 10.91 ± 3.99 μmol/l, *p* = 0.017). However, age at onset of cannabis use was not associated with alterations in plasma Hcy, folate and vitamin B12 (Table 5). Notably, lifetime diagnosis of cannabis abuse was not associated with BMI, psychopathological manifestation assessed by PANSS and the indices of cigarette smoking. However, there were significantly more males among FES patients with a lifetime diagnosis of cannabis abuse in comparison with the group of patients, who had never abused cannabis (90.47 vs. 31.42 %, *p* < 0.001).

**Table 4:** The comparison of first-episode schizophrenia patients with respect to lifetime diagnosis of cannabis abuse

	Patients with lifetime diagnosis of cannabis abuse (*N* = 21)	Patients without lifetime diagnosis of cannabis abuse (*N* = 35)	*p*-value^*^
Age (years)	23.81 ± 7.94	29.22 ± 4.50	0.102
Gender (males/females)	19/2	11/24	**<0.001** ^**†**^
BMI (kg/m^2^)	23.98 ± 3.24	23.41 ± 4.39	0.091
Glucose (mg/dl)	85.11 ± 8.85	86.08 ± 9.87	0.599
LDL (mg/dl)	88.08 ± 35.09	96.21 ± 29.77	0.479
HDL (mg/dl)	45.83 ± 18.56	55.10 ± 18.56	**0.041**
TC (mg/dl)	160.23 ± 47.31	173.00 ± 30.19	0.913
TG (mg/dl)	116.95 ± 73.87	110.09 ± 53.31	0.272
Hcy (μmol/l)	14.85 ± 7.35	10.91 ± 3.99	**0.017**
Folate (ng/ml)	5.14 ± 1.88	7.02 ± 3.12	0.425
Vitamin B12 (pg/ml)	382.48 ± 134.25	409.25 ± 235.44	**0.017**
The number of cigarette smokers	11	10	0.093
Pack-year index	2.26 ± 3.09	1.98 ± 4.00	0.746
Fagerström test	2.52 ± 2.87	1.54 ± 2.71	0.678
PANSS—positive symptoms score	25.09 ± 6.23	22.51 ± 4.50	0.101
PANSS—negative symptoms score	17.23 ± 7.75	18.08 ± 7.30	0.511
PANSS—general psychopathology score	43.85 ± 7.55	41.82 ± 7.81	0.265

*Abbreviations*: *BMI* body mass index, *Hcy* homocysteine, *HDL* high density lipoproteins, *LDL* low density lipoproteins, *PANSS* the Positive and Negative Syndrome Scale, *TC* total cholesterol, *TG* triglycerides

^*^ *p*-value refers to the comparison of mean-ranks using the Mann–Whitney *U*-test

^†^Significant differences after application of partial Bonferroni correction (*p* < 0.0054). Significant differences (*p* < 0.05) were marked in bold

**Table 5:** Correlations between plasma homocysteine, vitamin B12 and folate and selected clinical variables

	Homocysteine (μmol/l)	Vitamin B12 (pg/ml)	Folate (ng/ml)
DUP (days)	*r* = −0.272, ***p*** **= 0.042**	*r* = −0.068, *p* = 0.617	*r* = 0.168, *p* = 0.217
Age at onset of cannabis use (years)	*r* = 0.291, *p* = 0.242	*r* = −0.408, *p* = 0.093	*r* = −0.007, *p* = 0.977
PANSS—positive symptoms score	*r* = 0.093, *p* = 0.494	*r* = 0.031, *p* = 0.818	*r* = −0.222, *p* = 0.100
PANSS—negative symptoms score	*r* = 0.363, ***p*** **= 0.006**	*r* = −0.406, ***p*** **= 0.002**	*r* = −0.111, *p* = 0.415
PANSS—general psychopathology score	*r* = 0.349, ***p*** **= 0.008**	*r* = 0.013, *p* = 0.922	*r* = −0.365, ***p*** **= 0.006**

*Abbreviations*: *DUP* duration of untreated psychosis, *PANSS* Positive and Negative Syndrome Scale

Significant differences (*p* < 0.05) were marked in bold

Finally, we tested the hypothesis as to whether DUP and psychopathological manifestation of FES patients are associated with plasma levels of Hcy, folate and vitamin B12 (Table 5). We found that Hcy level predicted shorter DUP (*r* = −0.272, *p* = 0.042). Furthermore, Hcy level positively correlated with the severity of negative symptoms and general psychopathology assessed by PANSS (*r* = 0.363, *p* = 0.006 and *r* = 0.349, *p* = 0.008, respectively). In addition, plasma levels of vitamin B12 and folate negatively correlated with the severity of negative symptoms (*r* = −0.406, *p* = 0.002) and general psychopathology (*r* = −0.365, *p* = 0.006) in PANSS, respectively.

Differences in HDL and folate levels between FES patients and HC, as well as gender differences between FES patients with and without lifetime diagnosis of cannabis abuse remained significant after application of partial Bonferroni correction (Tables 1 and 4).

## Discussion

In this study, we have found that FES patients are characterized by higher plasma Hcy and lower levels of folate and HDL. These results cannot be attributed to differences in age, gender, BMI, cigarette smoking or antipsychotic treatments. Our findings are in agreement with previous studies showing higher levels of Hcy and lower levels of folate or vitamin B12 in first-episode psychosis patients (Garcia-Bueno et al. 2013; Ayesa-Arriola et al. 2012; Kale et al. 2010). Significantly lower levels of HDL are also in line with previous studies on FES patients (Wu et al. 2013; Phutane et al. 2011; Fleischhacker et al. 2013). Interestingly, in the European First-Episode Schizophrenia Trial (EUFEST), suboptimal HDL level was present in 28.5 % patients and was the most prevalent baseline metabolic risk factor (Fleischhacker et al. 2013).

Most interestingly, we have found that positive family history of schizophrenia in first and/or second degree relatives is associated with higher plasma Hcy, as well as with lower levels of HDL and vitamin B12. However, these results should be interpreted with caution as they were not significant after application of partial Bonferroni correction. This association has not been reported so far. The recent study by Geller et al. (2013) provided that siblings of patients with schizophrenia have higher Hcy levels than healthy controls. Simultaneously, there was no difference in Hcy level between schizophrenia patients and their siblings. Furthermore, there are studies showing that type 2 diabetes and obesity are more common in relatives of patients with schizophrenia (Mukherjee et al. 1989; Martins et al. 2001) or non-affective psychosis (Fernandez-Egea et al. 2008). These findings, along with those presented in this article, suggest that schizophrenia is in itself associated with metabolic dysregulation that originates from genetic susceptibility. On the basis of a meta-analysis, Muntjewerff (Muntjewerff et al. 2006) provided evidence that the C677T polymorphism in the *MTHFR* gene increases the risk of schizophrenia. Furthermore, there are studies showing that the C677T polymorphism in the *MTHFR* gene is associated with an earlier age of schizophrenia onset (El-Hadidy et al. 2013; Vares et al. 2010); however, some authors have not confirmed this finding (Saetre et al. 2011, 2012; Peerbooms et al. 2010). Finally, this polymorphism has been found to influence the development of metabolic syndrome components in response to antipsychotic treatment (Srisawat et al. 2013; Ellingrod et al. 2008, 2012; van Winkel et al. 2010a, b), cognitive performance (Kontis et al. 2013; Roffman et al. 2008a, b, 2011b), grey matter density (Zhang et al. 2013) and the activation of dorsal anterior cingulate cortex (Roffman et al. 2011a), the severity of aggressive behaviours (Dong et al. 2012) and negative symptoms (Roffman et al. 2008c, 2013a), as well as the efficacy of folate supplementation in the treatment of negative symptoms (Hill et al. 2011; Roffman et al. 2013b) in schizophrenia patients.

We have also found that more severe negative symptoms are associated with higher Hcy and lower vitamin B12 levels. These results are in line with previous studies showing that higher Hcy and lower folate levels are associated with higher severity of negative symptoms (Goff et al. 2004; Petronijevic et al. 2008; Bouaziz et al. 2010). However, there are also studies, which have failed to confirm this relationship (Neeman et al. 2005; Ma et al. 2009; Ayesa-Arriola et al. 2012). Inconsistent results might be due to recruitment of various subgroups of patients including first-episode schizophrenia-spectrum patients, acutely relapsed inpatients or chronic subjects. Notably, we have also found that higher Hcy and lower folate levels may predict more general psychopathology symptoms as assessed with the PANSS. These findings, along with the association with negative symptoms, have not been reported in FES patients so far. It should be noted that our results indicate an inverse relationship between plasma Hcy and DUP. The majority of previous studies have not confirmed this association (Petronijevic et al. 2008; Ma et al. 2009; Mabrouk et al. 2011). However, Ayesa-Arriola et al. (2012) found that higher Hcy levels may predict a shorter duration of untreated psychosis (DUP) in patients with first-episode schizophrenia-spectrum disorders. The exact mechanism of this association remains unknown as there is a scarcity of studies investigating the influence of antipsychotic treatment on Hcy level (Bicikova et al. 2011; Eren et al. 2010; Wysokinski and Kloszewska 2013). Moreover, these studies have provided contradictory results (Bicikova et al. 2011; Eren et al. 2010; Wysokinski and Kloszewska 2013). It might be hypothesized that a negative correlation between Hcy levels and DUP originates from more severe psychopathology in patients with higher Hcy levels since we have shown that Hcy level positively correlates with the severity of negative symptoms and general psychopathology assessed by PANSS. Therefore, more severe psychopathology due to high Hcy levels may shorten DUP.

We have also shown that a lifetime diagnosis of cannabis abuse is associated with higher Hcy, as well as lower HDL and vitamin B12 levels in FES patients. Notably, these differences were not significant after application of partial Bonferroni correction. We excluded current cannabis users with FES in order to increase the reliability of schizophrenia diagnosis. Notably, these results should be interpreted with caution, as we did not assess cannabis use in healthy controls. Furthermore, it might be feasible to examine one-carbon metabolism alterations in first-episode psychosis patients, who currently use cannabis. In the light of current knowledge, it is hard to indicate a putative linkage between cannabis use and Hcy metabolism. There are studies showing the association between cannabis use and low folate levels in pregnant women (Knight et al. 1994) and undergraduate students (Davis et al. 1978). These authors suggested that low folate level occurs most likely due to nutritional constraint. There are also studies showing that cannabinoids may impair the uptake of folate into BeWo cells (Araujo et al. 2009) and human syncytiotrophoblasts (Keating et al. 2009). However, the exact mechanism of this interaction remains unclear and requires further investigation.

Notably, our study has some limitations that may influence the results. These include sample size, the lack of quantitative assessment of cannabis use and evaluation of dietary intake, possible under-reporting bias of cannabis use within 1 year prior to the onset of psychosis and the influence of antipsychotic treatment. Although our sample was not large, it corresponds with sample sizes of previous studies on one-carbon metabolism deregulation in drug-naïve and/or FES patients (Bicikova et al. 2011; Kale et al. 2010; Bouaziz et al. 2010). Rates of under-reported illicit drug use in schizophrenia patients are considerable. For instance, in the recent study by Bahorik et al. (2013), more than half of patients did not report using illicit drugs. It is noteworthy that under-reporting bias seems to be undetectable in studies on lifetime use of illicit drugs. It cannot be ruled out that our results are confounded by the lack of evaluation of dietary intake. However, all subjects had BMI, folate and vitamin B12 levels within the normal range. Finally, we cannot exclude that our results are influenced by the use of antipsychotics. There is no doubt that antipsychotic treatment is strongly associated with the development of metabolic syndrome in schizophrenia (Hasnain et al. 2010). However, neither treatment duration nor chlorpromazine equivalent significantly influenced our results. Additionally, the observational study of drug-naïve schizophrenia patients by Bicikova et al. (2011) did not reveal significant changes in Hcy level after a 6-month follow-up. Similarly, the cross-sectional study by Wysokinski and Kloszewska (2013) did not reveal the influence of clozapine on Hcy level. There is only one study (Eren et al. 2010) on chronic schizophrenia patients showing that higher doses of typical antipsychotics (CPZ equivalent > 400 mg) might be associated with lower levels of plasma folate, but not Hcy or vitamin B12.

In conclusion, it should be noted that metabolic alterations seem to be more common in FES patients. It is most likely that schizophrenia is in itself linked to metabolic disturbances that have strong genetic underpinnings. Alterations in one-carbon metabolism seem to be associated with FES psychopathology. An important but preliminary finding from our study, is the long-term deleterious effect of cannabis use on metabolic profile. The influence of various clinical factors on the severity of one-carbon metabolism dysfunction warrant the need of a personalized medicine approach in the treatment of psychopathological symptoms of schizophrenia and comorbid early deleterious alterations in metabolic profile. The results of our study could provide additional support to the ‘chicken–egg dilemma’ with respect to metabolic alterations in schizophrenia. The majority of studies focus on the fact that antipsychotic treatment increases the risk of metabolic disturbances in the course of schizophrenia; however, it should be stressed that there are also metabolic alterations that occur prior to the onset of psychosis and that are more common among patients having relatives with schizophrenia. Such observations should lead to further research projects looking more closely into bidirectional relationships between biochemical metabolic markers and schizophrenia.
